# Laparoscopic proximal gastrectomy with double-tract reconstruction for upper third gastric cancer

**DOI:** 10.1186/s12893-021-01153-y

**Published:** 2021-03-19

**Authors:** Shuo-meng Xiao, Ping Zhao, Zhi Ding, Rui Xu, Chao Yang, Xiao-ting Wu

**Affiliations:** 1 Department of Gastrointestinal Surgery, West China School of Medicine, Sichuan University, Chengdu, Sichuan China; 2 Department of Gastrointestinal Surgery, Sichuan Cancer Hospital, Chengdu, China

**Keywords:** Gastric cancer, Double-tract reconstruction, Open proximal gastrectomy, Laparoscopy proximal gastrectomy, Safety, Feasibility

## Abstract

**Background:**

Proximal gastrectomy with double-tract reconstruction (DTR) has been used for upper third gastric cancer as a function-preserving procedure. However, the safety and feasibility of laparoscopic proximal gastrectomy (LPG) with DTR remain uncertain. This study compared open proximal gastrectomy (OPG) with DTR and LPG with DTR for proximal gastric cancer.

**Methods:**

Sixty-four patients who had undergone OPG with DTR and forty-six patients who had undergone LPG with DTR were enrolled in this case–control study. The clinical characteristics, surgical outcomes and postoperative nutrition index were analysed retrospectively.

**Results:**

The operation time was significantly longer in the LGP group than in the OPG group (258.3 min vs 205.8 min; *p* = 0.00). However, the time to first flatus and postoperative hospital stay were shorter in the LPG group [4.0 days vs 3.5 days (*p* = 0.00) and 10.6 days vs 9.2 days (*p* = 0.001), respectively]. No significant difference was found between the two groups in the number of retrieved lymph nodes, complications or reflux oesophagitis. The nutrition status was assessed using the haemoglobin, albumin, prealbumin and weight levels from pre-operation to six months after surgery. No significant difference was found between the groups.

**Conclusion:**

LPG with DTR can be safely performed for proximal gastric cancer patients by experienced surgeons.

## Introduction

The incidence of proximal gastric cancer is increasing [1, 2]. The rate of oesophagogastric junctional adenocarcinoma has risen from 22.3% to 35.7% in the last twenty years [3]. For early-stage proximal gastric cancer, total gastrectomy and proximal gastrectomy are options. The benefits of total gastrectomy are lymph node dissection of the distal stomach and reduction of gastroesophageal reflux. However, dystrophia can occur. Although total gastrectomy is considered the standard procedure, proximal gastrectomy as a function-preserving procedure is accepted by some doctors. However, proximal gastrectomy using traditional oesophagogastrostomy is associated with gastroesophageal influx and anastomotic stricture [4]. Function preservation and anti-reflux are the same important concerns.

Double-tract reconstruction (DTR) was first introduced in 1988 [5] and can reduce the rate of reflux oesophagitis. Recent studies have shown that double-tract reconstruction is superior to total gastrectomy [6, 7]. Compared with total gastrectomy, proximal gastrectomy is less invasive and preserves the distal stomach. A retrospective study found that the 5-year overall survival was not different between proximal gastrectomy with DTR and total gastrectomy with Roux-en-Y [8]. Proximal gastrectomy with DTR is a good choice for proximal gastric cancer patients.

Laparoscopic distal gastrectomy has been used to treat distal gastric cancer [9, 10]. For upper third gastric cancer, laparoscopic proximal gastrectomy (LPG) is performed as a surgical option. Compared with open proximal gastrectomy (OPG) with DTR, LPG with DTR is a complicated procedure, and its clinical use is limited. This study investigated the safety and feasibility of this procedure.

## Methods

### Patients

From August 2015 to March 2020, OPG with DTR or LPG with DTR was performed at our hospital. The inclusion criteria for all the patients who had undergone proximal gastrectomy with DTR were as follows: (1) a diagnosis of proximal gastric adenocarcinoma, (2) a distal edge of the tumour less than 3 cm under the cardia, (3) a proximal edge of the tumour less than 1 cm above the cardia, and (4) a preoperative clinical stage of T_1-3_N_0-1_M_0_ according to the 7th and 8th editions of the American Joint Committee on Cancer [11]. This study protocol received approval from the Institutional Review Board of Sichuan Cancer Hospital.

### Surgical procedure

The operations were performed by two surgical teams. The two surgeons had performed more than 100 laparoscopic gastrectomies and 50 open proximal gastrectomies with DTR.

### LPG with DTR

**Fig. 1:**
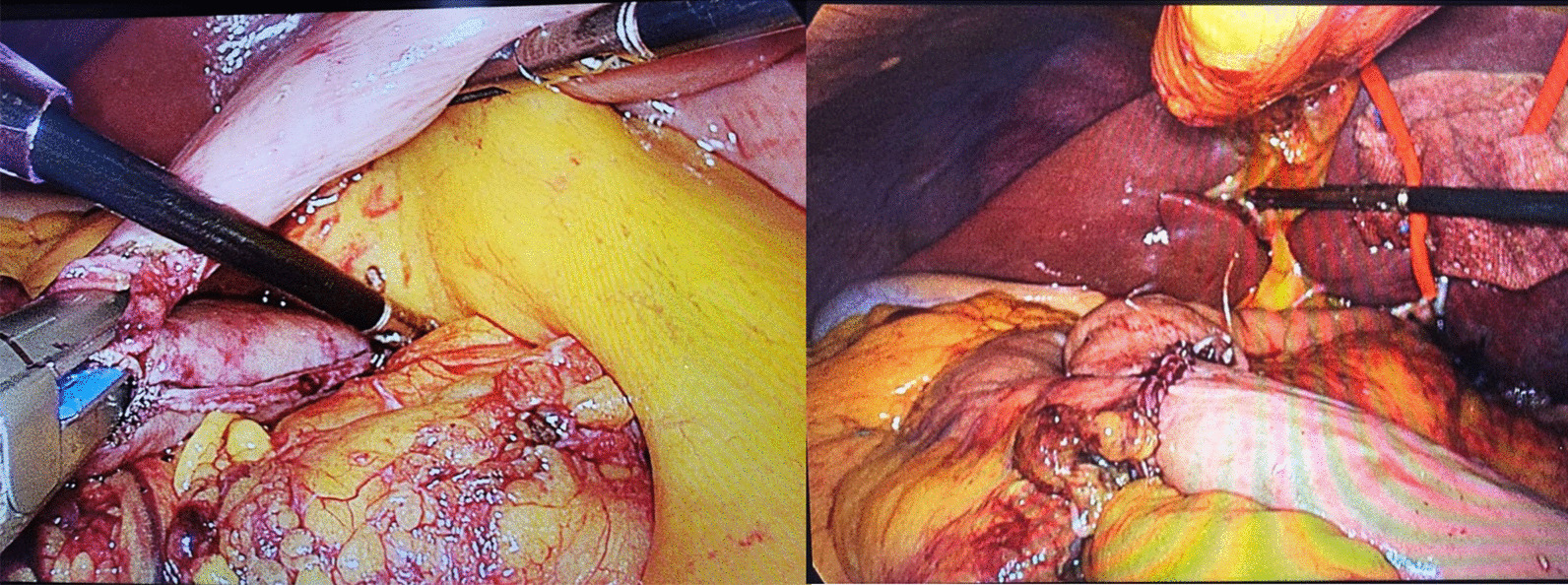
Proximal gastrectomy with DTR

LPG and D1 + lymphadenectomy were performed according to the 2014 Japanese Gastric Cancer Treatment Guidelines (version 4) [12]. Briefly, lymph nodes no. 1, 2, 3a, 4sa, 4sb, 7, 8a, 9, and 11p were dissected. The stomach and oesophagus were transected using endoscopic linear staplers. A negative surgical margin and a large remnant stomach were obtained. An approximately 3- to 4-cm umbilical trocar incision was extended to remove the specimen. The jejunum 20 cm distal from the ligament of Treitz was transected using an endoscopic linear stapler; the first side-to-side oesophagojejunostomy was also performed using an endoscopic linear stapler. The second side-to-side gastrojejunostomy was performed at the point 10–15 cm distal from the first anastomosis. The second anastomosis was made on the front wall and greater curvature of the remnant stomach (Fig. 1). The third side-to-side jejunojejunostomy was performed 20–25 cm below the second anastomosis. These common openings were sutured using a 3–0 barbed suture. In this group, a drainage tube was used in all patients. On postoperative day (POD) 2, the patients were allowed to drink water. A liquid diet was ingested on POD 3 to 4, and regular food was ingested on POD 6 to 7.

### OPG with DTR

OPG and D1 + lymphadenectomy were performed. Lymph nodes no. 1, 2, 3a, 4sa, 4sb, 7, 8a, 9, and 11p were dissected. The stomach was transected using a linear stapler. The oesophagus was cut using an operating knife. The jejunum 20 cm distal from the ligament of Treitz was transected using an operating knife. The first side-to-end oesophagojejunostomy was performed using a circle stapler. Next, the second side-to-end gastrojejunostomy was performed using the circle stapler 10–15 cm distal from the first oesophagojejunostomy. An anastomosis was made on the front wall and greater curvature of the remnant stomach. The third side-to-end jejunojejunostomy was performed using a circle stapler 20–25 cm distal to the second gastrojejunostomy. In this group, the postoperative management protocol was the same as that in the LPG group. A drainage tube was used in all the patients. On postoperative day (POD) 2, the patients were allowed to drink water. The liquid diet was ingested on POD 3 to 4, and regular food was ingested on POD 6 to 7.

### Clinical outcomes

Complications were classified using the Clavien-Dindo classification system [13]. Sex, age, body mass index (BMI), Patient-Generated Subjective Global Assessment (PG-SGA), American Society of Anaesthesiologists score for physical status (ASA-PS), pathological stage and nutrition index were collected retrospectively. The following surgical outcomes were obtained: operation time, lymphadenectomy type, retrieved lymph nodes, time to first flatus, postoperative hospital stay, and complications. Nutrition indexes, including the haemoglobin, albumin, prealbumin and weight levels, were collected at one month and 6 months after the operation. For all the patients, endoscopy was performed once a year after the operation. Reflux oesophagitis was graded according to the Los Angeles classification.

### Statistical analysis

The SPSS program (version 26; IBM Corporation, USA) was used for statistical analysis. Chi-squared test was employed for categorical variables, and Student’s t test was employed for continuous variables. A *P* value less than 0.05 was considered statistically significant.

## Results

### Patient characteristics

The characteristics of the patients who had undergone OPG with DTR and LPG with DTR are detailed in Table 1. All the included cases were classified as Siewert II gastroesophageal junction cancers. No significant differences were found in the sex, age, PG-SGA, ASA-PS or pathological stage. The BMI was higher in the OPG group than in the LPG group (*p* = 0.01).

**Table 1:** Characteristic of patients

	OPG with DTR(64)	LPG with DTR (n = 46)	*P* value
Sex			0.44
Male	49	1538	
Female	3815	8	
Age	66.3 ± 9.0	65.9 ± 7.4	0.80
BMI	23.9 ± 3.2	22.5 ± 2.5	0.01
PG-SGA	4.9 ± 4.3	6.5 ± 5.0	0.08
ASA-PS			0.60
1	7	8	
2	52	34	
3	5	4	
Pathological stage			0.26
IA	14	7	
IB	16	11	
IIA	26	26	
IIB	8	2	

*LPG* Laparoscopic proximal gastrectomy, *OPG* Open proximal gastrectomy, *DTR* Double-tract reconstruction

### Surgical outcomes and complications

The surgical outcomes and complications are detailed in Table 2. The operation time was significantly longer in the LPG group (258.3 min) than in the OPG group (205.8 min). However, the time to first flatus was shorter in the LPG (4.0 vs 3.5, *p* = 0.00). In terms of retrieved lymph nodes, no significant difference was found between the groups. However, the length of postoperative hospital stay was significantly shorter in the LPG group (9.2 vs 10.6; *p* = 0.001).

**Table 2:** Surgical outcomes and complications

	OPG with DTR (n = 64)	LPG with DTR (n = 46)	*P* value
Preoperative ESD	0	2	0.17
Operation time, minutes	205.8 ± 45.0	258.3 ± 58	< 0.001
Lymphadenectomy	D1 +	D1 +	
Retrieved lymph nodes	19.7 ± 8.7	19.2 ± 3.1	0.67
Time to first flatus, days	4.0 ± 0.7	3.5 ± 0.6	< 0.001
Postoperative hospital stay	10.6 ± 1.3	9.2 ± 2.6	0.001
Complications (Clavien-Dindo classification grade ≤ II)			
Uroschesis	0	1	0.42
Chylous fistula	2	1	1.0
Pancreatic leakage	1	1	0.42
Anastomotic stricture	1	0	1.0
Pneumonia	7	5	0.99
Intra-abdominal infection	2	1	1.0
Anastomotic leakage	0	1	0.42
Complications (total)	13 (20.3%)	10 (21.7%)	0.86

*OPG* open proximal gastrectomy, *LPG* laparoscopic proximal gastrectomy, *DTR* double-tract reconstruction, *ESD* Endoscopic submucosal dissection

In the OPG group, twelve patients had thirteen complications (20.3%). One patient had chylous fistula and intra-abdominal infection. In the LPG group, ten patients had complications (23.3%). One of these patients had anastomotic leakage, which was treated with nutrition therapy and then was cured on the postoperative twenty-fifth day. These complications in both groups were classified as grade ≤ II. No significant differences were found between the groups in the complication rates for uroschesis, chylous fistula, anastomotic stricture, pneumonia, intra-abdominal infection and anastomotic leakage (*p* > 0.05). Forty-six patients in the OPG group and thirty-four in the LPG group had undergone endoscopy after surgery. Reflux oesophagitis was found in five patients (10.9%) in the OPG group and four (11.7%) in the LPG group (Table 3). No significant difference was found between the groups (*p* = 1.0).

**Table 3:** Rate of endoscopic reflux esophagitis after surgery

Reflux esophagitis^a^	OPG with DTR (n = 46)	LPG with DTR (n = 34)	P value
Grade A	4	3	
Grade B	1	1	
Grade C	0	0	
Grade D	0	0	
	10.9%	11.7%	1.0

*LPG* Laparoscopic proximal gastrectomy, *OPG* Open proximal gastrectomy, *DTR* Double-tract reconstruction

^a^Reflux esophagitis according to the Los Angeles classification

### Nutrition index

**Fig. 2:**
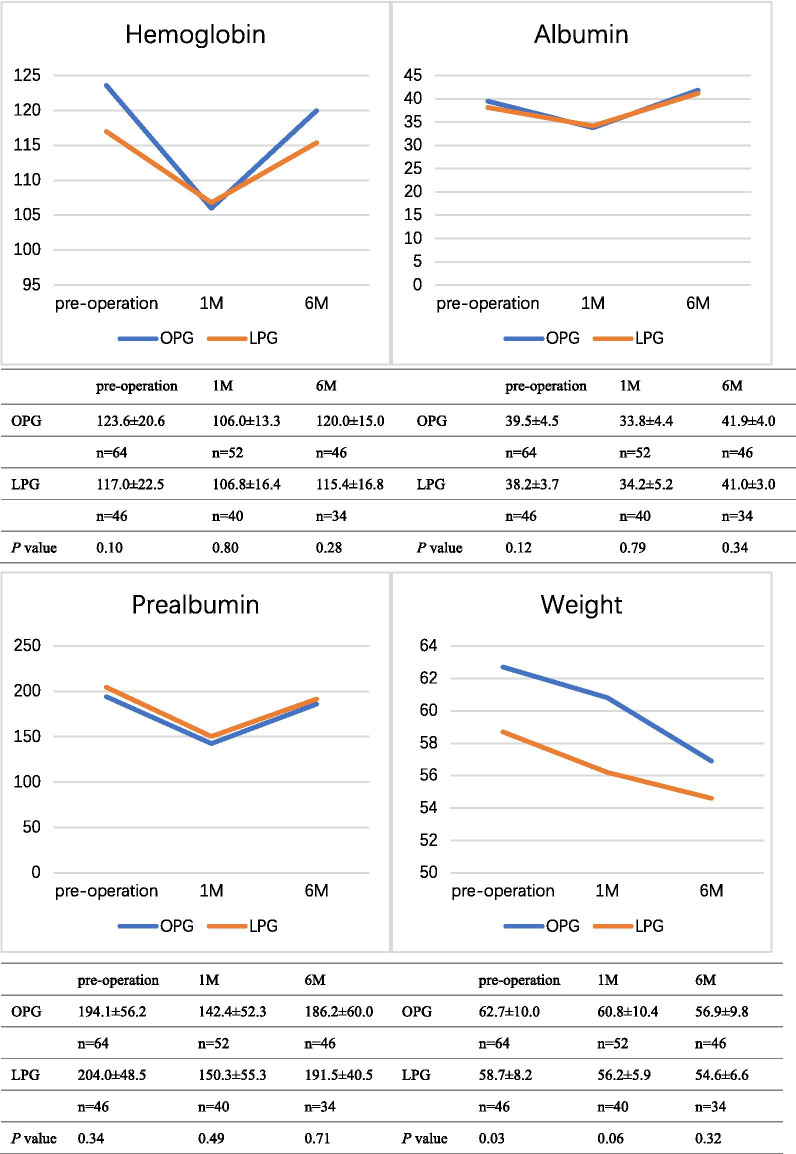
Comparison of the nutrition indexes between OPG with DTR and LPG with DTR. OPG: open proximal gastrectomy; LPG: laparoscopic proximal gastrectomy; 1 M: 1 month after surgery; 6 M: 6 months after surgery

Nutrition was compared between the groups by assessing the haemoglobin, albumin, prealbumin and weight levels (Fig. 2). No significant difference was found between the groups in the nutrition indexes before surgery. After surgery, the haemoglobin, albumin, prealbumin and weight levels declined in both groups, although no significant difference was observed one month or six months after surgery (*p* > 0.05).

## Discussion

The incidence of proximal gastric cancer has increased in the last twenty years[3]. For early-stage proximal gastric cancer, radical operation and functional preservation are the same concerns. Proximal gastrectomy for selected patients is an option. However, because of reflux oesophagitis and anastomosis stricture, traditional oesophagogastrostomy is gradually not being used. Jejunal interposition reconstruction can avoid these complications, and a prospective study found that DTR may be suitable for patients with impaired glucose tolerance [14]. Meta-analysis results have revealed that proximal DTR is superior to total gastrectomy concerning reflux syndrome and the nutritional status [15, 16]. DTR has been accepted by some doctors. This reconstruction can preserve the distal stomach and reduce reflux oesophagitis [7, 17]. The present study found that its safety and feasibility were equal to those of OPG with DTR, although the operation time was longer in the LPG group.

Compared with proximal gastrectomy, total gastrectomy can dissect lymph nodes no. 5, 6, and 12. A large-scale study showed that the rate of lymph node no. 5 metastasis was zero for T1 patients and 0.5% for T2 patients, and the rate of lymph node no. 6 metastasis was 0.1% for T1 patients and 0.9% for T2 patients [18]. The diameters of the tumours in our study were less than 4 cm. A retrospective study found that the distance from the oesophagogastric junction to the distal end of the tumour decides between proximal gastrectomy and total gastrectomy [19]. When the distance was ≤ 30 mm, the rate of node metastasis along the greater curvature or antrum was 2.2%, and proximal gastrectomy was safe. When the distance was > 50 mm, the rate of node metastasis along the greater curvature or antrum was 20%, and total gastrectomy was necessary.

According to another study, the overall 5-year survival rates for patients who had undergone proximal gastrectomy were higher than those for patients who had undergone total gastrectomy [20]. Based on the present study, proximal gastrectomy is feasible.

For proximal gastrectomy, the reported anastomosis methods included oesophagogastrostomy, jejunal interposition and DTR [5, 21, 22]. Laparoscopic proximal gastrectomy using the double-flap technique is a novel method to reduce reflux oesophagitis and anastomosis stricture [23], although this reconstruction is a complicated procedure and requires a longer operation time (386.5 min) [21]. However, the operation time of LPG with DTR ranges from 198.3 min to 268.2 min [6, 24, 25], as shown in our study (258.3 min). Although the operation time was shorter in the OPG group (205.8), LPG had the advantage of a shorter postoperative recovery. The time to first flatus and postoperative hospital stay was also shorter in the LPG group, and these differences were statistically significant. In actual clinical work, this approach might lead to a more satisfying medical experience and save medical resources. In previously reported studies, the morbidity rate of LPG with DTR ranged from 9.6% to 32.3% [6, 24–26]. In our study, the rate of complications was 21.7% in the LPG group, the same as that in the OPG group (20.3%). The rate of reflux oesophagitis was 11.7% in the LPG group and 10.9% in the OPG group. This result was the same as that in a previous study [27]. More patients and longer follow-up periods are required to verify the findings.

Previous studies have reported the nutritional benefits of LPG over laparoscopic total gastrectomy [27]. In our study, no difference was found in the nutrition status between the groups; thus, LPG with DTR or OPG with DTR maintains the nutrition status. Previous studies have revealed that if food cannot flow into the remnant stomach, DTR is the same as total gastrectomy. When most food flows into the remnant stomach, DTR has benefits [28]. Theoretically, a 60-mm linear stapler can produce a larger anastomosis than a circular stapler. However, no difference was found in the nutrition index between the groups. Large-scale studies and long-term follow-up are required to confirm the results. Anastomosis was also performed on the front wall and greater curvature of the remnant stomach, and long-term follow-up is necessary.

## Conclusions

Our findings indicate that LPG with DTR can be safely performed by experienced surgeons in proximal gastric cancer patients.

## Data Availability

The datasets used and/or analysed during the current study are available from the corresponding author on reasonable request.
